# Effect of Natural Antioxidants from Marigolds (*Tagetes erecta* L.) on the Oxidative Stability of Soybean Oil

**DOI:** 10.3390/molecules27092865

**Published:** 2022-04-30

**Authors:** Xiuqiong Huang, Wei Gao, Xuan Yun, Zhixing Qing, Jianguo Zeng

**Affiliations:** 1 Hunan Key Laboratory of Traditional Chinese Veterinary Medicine, Hunan Agricultural University, Changsha 410128, China; huangxqiong@163.com (X.H.); 15674853170@163.com (X.Y.); 2 College of Food and Chemical Engineering, Shaoyang University, Shaoyang 422000, China; 3 College of Horticulture, Hunan Agricultural University, Changsha 410128, China; 4 Chenguang Biotechnology Company Limited, Handan 056000, China; gaowei2003425@163.com

**Keywords:** *Tagetes erecta* L., quercetagetin, DPPH-HPLC-Q-TOF/MS, HS-SPME-GC-MS, oxidative stability

## Abstract

In recent years, synthetic antioxidants that are widely used in foods have been shown to cause detrimental health effects, and there has been growing interest in antioxidants realised from natural plant extracts. In this study, we investigate the potential effects of natural antioxidant components extracted from the forage plant marigold on the oxidative stability of soybean oil. First, HPLC-Q-TOF-MS/MS was used with 1,1-diphenyl-2-picrylhydrazyl (DPPH) to screen and identify potential antioxidant components in marigold. Four main antioxidant components were identified, including quercetagetin-7-*O*-glucoside (1), quercetagetin (2), quercetin (3) and patuletin (4). Among them, quercetagetin (QG) exhibited the highest content and the strongest DPPH radical scavenging activity and effectively inhibited the production of oxidation products in soybean oil during accelerated oxidation, as indicated by reductions in the peroxide value (PV) and acid value (AV). Then, the fatty acids and volatile compounds of soybean oil were determined with gas chromatography–mass spectrometry (GC-MS) and headspace solid-phase microextraction–gas chromatography–mass spectrometry (HS-SPME-GC-MS). A total of 108 volatile components, including 16 alcohols, 23 aldehydes, 25 ketones, 4 acids, 15 esters, 18 hydrocarbons, and 7 other compounds, were identified. QG significantly reduced the content and number of aldehydes and ketones, whereas the formation of acids and hydrocarbons was completely prevented. In addition, the fatty acid analysis demonstrated that QG significantly inhibited oxidation of unsaturated fatty acids. Consequently, QG was identified as a potential, new natural antioxidant that is believed to be safe, effective and economical, and it may have potential for use in plant extracts feed additives.

## 1. Introduction

Interest in safe and natural antioxidants is currently increasing the use of plant extracts feed additives to serve as antioxidants in animal nutrition. Consumers are interested in safe and natural foods of animal origin, and in some cases, they are also willing to pay a premium price for them [1]. Moreover, the European Union has banned in-feed antibiotics in animal nutrition to prevent antibiotic-resistant bacteria, which has increased interest in the use of plant extracts feed additives as dietary ingredients in animal nutrition [2]. Moreover, the possibility of using plant extracts feed additives, such as essential oils, plant extracts, and in particular, plant food industry by-products, is of great interest since it reduces industry wastes and is in harmony with the philosophy underlying environmental sustainability [3]. Marigold (*Tagetes erecta* L.) is a natural forage plant and an annual herb of the genus Tagetes in the family Asteraceae; it is also known as Hibiscus and is native to Mexico and South America [4]. It is widely cultivated in China for its strong weather resistance, wide adaptability, long flowering period, varied flowers and pure flower colours. It can be used as an ornamental flower, medicinal plant and food spice [5,6]. Studies have shown that marigold contains a variety of bioactive components, including carotenoids [7], flavonoids [8], phenolic acids [9] and thiophenes [10]. Additionally, marigolds have been shown to have a range of pharmacological activities, including antioxidant [11,12], vision protection [13], anti-inflammatory [14], diuretic [15] and antibacterial [16] activities. Marigold residue and marigold powder are widely used as feed ingredients. Xanthophyll pigments from marigold flowers, including zeaxanthin and lutein, have been used as food additives and food colouring agents [17]. Intriguingly, lutein, the main pigment in marigold, has been used as an antioxidant, a food colouring agent and a nutraceutical [18], and it also effectively reduces the chance of developing macular degeneration, cataracts, atherosclerosis and certain cancers [19]. In particular, antioxidants such as the macular carotenoids lutein, zeaxanthin and meso-zeaxanthin possess significant antioxidant and anti-inflammatory effects in the retina [20]. In addition to lutein, marigolds contain many other active bioactive components that can be exploited [7]. Therefore, the development of potential antioxidants from marigold into plant feed additives is an economically important goal for the high-value utilization of marigolds.

Conventional bio-guided fractionation for the discovery of active ingredients from natural products generally employs a series of isolation and purification techniques for obtaining monomeric components, followed by in vivo or in vitro pharmacological screening, and this method plays an important role in drug development; however, it is a time-consuming, laborious and costly task [21,22]. Additionally, the activities of some natural ingredients often decrease during isolation and purification due to the breakdown of components [23]. Offline and online DPPH-HPLC-Q-TOF-MS/MS are suitable for achieving efficient screening and identification of bioactive compounds from natural product extracts [24]. Therefore, a rapid and efficient DPPH free radical spiking test was explored to screen antioxidants from marigold.

The oil composition in feed is the main energy source of animal nutrient intake. The presence of rancid flavour in the feed means that it has been oxidatively spoiled, which is mainly indicated by oxidative rancidity involving unsaturated bonds of lipids [25]. Soybean oil is the one of the most heavily consumed vegetable oils worldwide and has been thought to aid the absorption of some liposoluble nutrients while also reducing feed dust and increasing feed energy [26]. However, high-temperature heating causes uncontrolled oxidation of highly unsaturated oils, triggering a series of complex chemical reactions that lead to the formation of various oxidation products, such as acids, aldehydes and ketones, and ultimately, to lipid peroxidation [27,28,29]. Oxidation of vegetable oil results in a significant decrease in the nutritionally mostly beneficial polyunsaturated fatty acids, while the contents of trans isomers and saturated fatty acids exhibiting unfavourable nutritional effects are significantly increased [30,31]; this eventually causes adverse physiological reactions [32] and reduces the palatability of the ration, which affects animal feeding and can lead to refusal to eat, post-feeding poisoning or even death [33,34]. Thus, in order to retard or inhibit oxidative processes and increase the health aspects and nutritional value of oil, some synthetic antioxidants, such as butylated hydroxyanisole (BHA), hydroxytoluene (BHT), propyl gallate (PG), tert-butylhydroquinone (TBHQ) and ethoxyquin (EQ), have been used to delay the oxidative deterioration of oil during storage [35,36], but synthetic antioxidants have come under scrutiny due to potential toxicological effects and human health risks. Therefore, the use of synthetic antioxidants has been restricted [37]. Natural products are again the focus of attention, including useful and natural plant-based antioxidants, such as tea extracts [38], rosemary extracts [39] and curcumin [40], which are used for food protection and disease prevention. Therefore, research on safe and effective natural antioxidants from natural sources, such as phenolic compounds, is being explored to find antioxidants capable of protecting fats and oils from oxidation [41].

QG is a bioactive component found in the forage plant marigold, where it exhibits high content and strong antioxidant capacity. Its antioxidant capacity can be exploited in the feed industry to maintain feed quality by using marigold as a source of antioxidants that prevent rancidity and the oxidation of lipids. However, no studies have been conducted to determine the efficacy of QG in preventing lipid oxidation under accelerated oxidation conditions. Therefore, we investigated the effectiveness of different QG concentrations (100, 200, 400, 800 and 1600 ppm) in protecting soybean oil from accelerated oxidation and compared the antioxidant strength with those of the synthetic antioxidants BHT and PG and the natural antioxidant TP under the same conditions.

The aim of the present study was to explore the effects of the strongest radical scavenging antioxidants found in marigold on the oxidative stability of soybean oil during accelerated oxidation. This study used an offline DPPH-HPLC-Q-TOF/MS system to identify potentially optimal antioxidants and to evaluate their effectiveness in retarding lipid oxidation in soybean oil by measuring the acid value, peroxide value, fatty acid composition and volatiles composition during accelerated oxidation storage. The results of this study indicated that QG is the best antioxidant among four antioxidant components identified in marigolds, and it significantly improved lipid stability during the accelerated oxidation of soybean oil. Therefore, the study suggested that QG has great potential for use as a natural antioxidant in feed additive products.

## 2. Results

### 2.1. Screening of Potential Antioxidant Components in Methanol Extracts of Marigold

**Figure 1:**
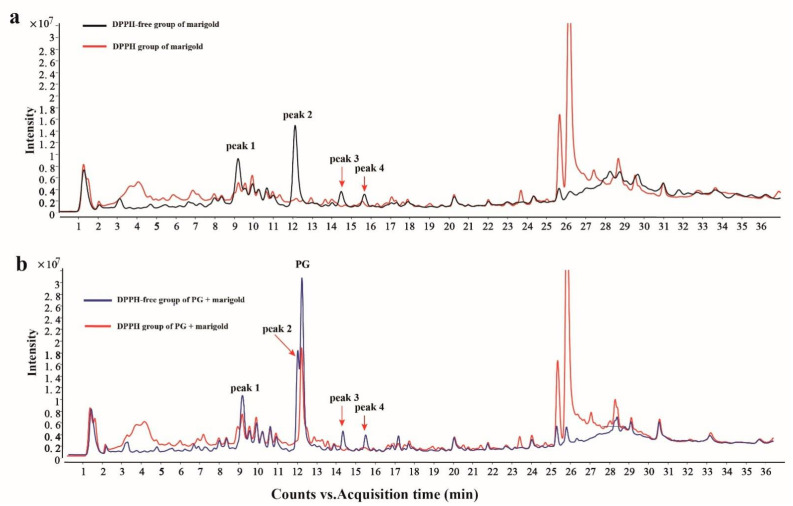
Chromatograms of the methanol extract of marigold before and after reaction with DPPH radicals (**a**); chromatograms of the methanol extract of marigold with PG chromatogram before and after reaction with DPPH radicals (**b**).

To rapidly screen the antioxidant components present in marigolds, an offline DPPH-HPLC-Q-TOF/MS method was used. Under optimized HPLC conditions, four main compounds of marigold were detected and isolated with retention times of 9.262 min (quercetagetin-7-O-glucoside), 12.144 (quercetagetin), 14.547 (quercetin) and 15.748 (patuletin) (Figure 1a). The strength of the antioxidant activity was judged by analysing the extent of the reduction in the peak area in the HPLC chromatogram after the active antioxidant components reacted with DPPH free radicals. In addition, to investigate the feasibility of the method, the results were calibrated with a PG positive control, and the retention time of the PG peak was 12.319, as shown in Figure 1b, and the peak area for PG was significantly reduced by 28.36% after reaction with DPPH. The data indicated that PG has a strong free radical scavenging ability and is a recognized antioxidant, further validating the feasibility of the offline DPPH-HPLC-Q-TOF/MS method. To evaluate the free radical scavenging ability of the four antioxidant components corresponding to peaks 1, 2, 3 and 4, the marigold extract was reacted with DPPH at different concentrations and then analysed by HPLC. With the peak areas (PAs) of peaks 1, 2, 3 and 4 in the untreated sample set as 100%, the relative PAs were calculated for the extract samples reacted with DPPH at concentrations of 0.625 mM, 1.25 mM, 2.5 mM and 5 mM, and the results are shown in Table 1. It was found that the areas of all four peaks decreased with increasing DPPH concentration. The peak 2 (quercetagetin) area decreased more sharply than those of the other three peaks. It was deduced that the antioxidant quercetagetin exhibited the greatest radical scavenging capacity.

### 2.2. Identification of Screened Antioxidants in the Methanol Extract of Marigold

**Figure 2:**
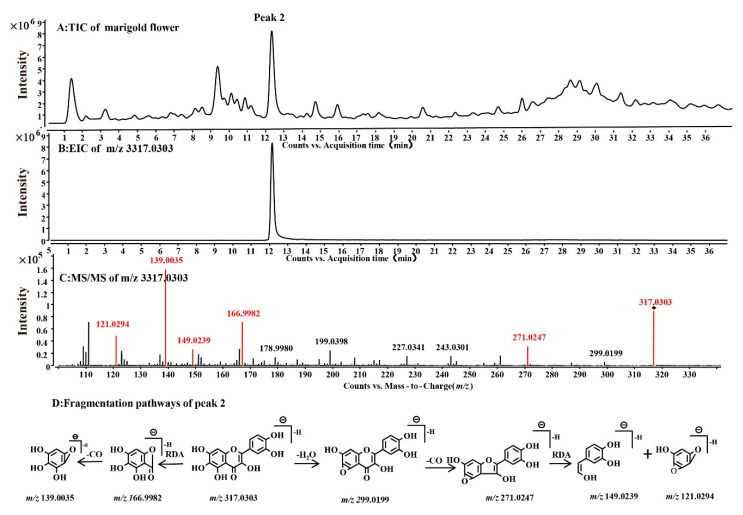
Screening of compound 2 by accurate-target means in the TIC (**A**), EIC (**B**) and MS/MS spectra (**C**) and the fragmentation pathway of compound 2 in the methanol extract of marigold flower (**D**).

Potential antioxidant compounds were detected via offline DPPH-HPLC-Q-TOF/MS. The molecular weights of the four antioxidant compounds were determined by Q-TOF-MS/MS in negative ion mode, and their MS/MS information are shown in Table 2. Compound 2 was used as an example for mass spectrometry analysis. The EIC of the theoretical mass (*m*/*z* 317.0303, [M-H]-) of a reported compound in the TIC of the flower (Figure 2A) was determined. The result showed that Compound 2 appeared in high abundance (Rt 12.144 min), and its measured MS fragmentation negative mode was *m*/*z* 317.0303 (δ ppm: 1.57) (Figure 2B). The MS/MS spectrum of this compound (Figure 2C) suggests that the first fragmentation pathway produced the fragment by the retro-Diels–Alder (RDA) reaction, and the fragments were *m*/*z* 166.9982 and 149.0239. The second fragmentation pathway involved the loss of the small molecules CO and H_2_O from the deprotonated skeleton, and the ions at *m*/*z* 139.0036 were formed by the neutral loss of CO moieties from the precursor ions at *m*/*z* 166.9982. Compound 2 was preliminarily identified as QG by analysing its mass spectra and references [32] (Figure 2D). Similarly, by analysing the MS spectra (Figure S1), peak 1, 3 and 4 were preliminarily identified as corresponding to quercetagetin-7-*O*-glucoside (1), quercetin (3) and patuletin (4).

### 2.3. Quantitative Analysis of Quercetagetin-7-O-Glucoside, QG and Quercetin in Methanol Extracts of Marigolds

Quantitative analyses of the main natural antioxidants, including quercetagetin-7-*O*-glucoside (1), quercetagetin (2) and quercetin (3), were carried out with an external standard method. Calibration curves were constructed from peak areas as a function of compound concentrations. The three reference compounds were eluted at 6.615, 8.104 and 9.718 min, respectively, as seen in the chromatogram (Figure S2a). In the marigold extract sample, the three compounds were eluted at the same retention time and exhibited baseline separation from other components in the sample (Figure S2b). Calibration curves as well as correlation coefficients (R2) and linear ranges are listed in Table S2. The contents of quercetagetin-7-*O*-glucoside (1), quercetagetin (2) and quercetin (3) were calculated, and the average contents were 31.02 ± 0.96 µg/mg, 65.18 ± 2.08 ug/mg and 0.46 ± 0.05 µg/mg. The data showed that quercetagetin exhibited the highest content among the antioxidant components in marigold.

### 2.4. Changes in Peroxide Value and Acid Value of Quercetagetin (QG) during Accelerated Oxidation of Soybean Oil

**Figure 3:**
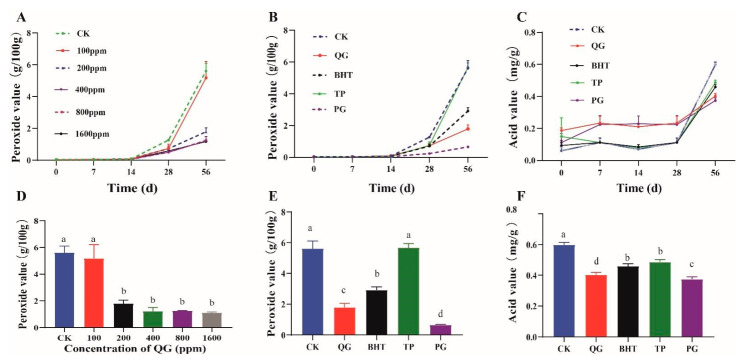
Mean peroxide value (PV) for pure soybean oil and oil treated with different concentrations (100, 200, 400, 800 and 1600 ppm) QG (**A**); mean peroxide value (PV) for pure soybean oil, QG and positive antioxidants with 200 ppm (**B**); mean acid value (AV) for pure soybean oil, QG and positive antioxidants with 200 ppm (**C**); mean PV in soybean oil treated with different concentrations QG after 56 days (**D**); mean PV in soybean oil treated with different antioxidants after 56 days (**E**); mean AV in soybean oil treated with different antioxidants after 56 days (**F**). Error bars represent the mean value ± SD. Significant differences between diet groups at the indicated week are signified by letters, where different letters indicate difference (*p* < 0.05) between groups, while the same letter indicates no difference.

The peroxide value (PV) was used to measure the degree of primary oxidation of soybean oil after five different periods (0, 7, 14, 28 and 56 days). As shown in Figure 3A, increases in the accelerated oxidation time and the concentration of added QG resulted in stronger inhibitory effects. Interestingly, the oils with 200, 400, 800 and 1600 ppm QG showed lower PVs than the pure oil (CK). When the oil was treated with 100 ppm QG, the PV showed no significant difference compared with that for pure oil. Therefore, we speculate that 200 ppm QG in soybean oil exhibits very good antioxidant effects. As shown in Figure 3D, on the 56th day of accelerated oxidation, the measured PV showed that the addition of higher concentrations of QG to soybean oil led to lower PVs and stronger antioxidant effects, which indicated that the antioxidant capacity of QG with soybean oil was related to the concentration of QG. Additionally, the PV data showed that the sample treated with 1600 ppm QG was most affected, with approximately 8.5 times the level of lipid peroxidation compared to the pure oil, which indicated that QG prolonged the initiation stage of soybean oil oxidation. As shown in Figure 3B, a comparison of the three positive controls with the extension of accelerated oxidation time showed that the PV did not significantly change from 0 to 14 d, and it gradually increased after 14 d. As shown in Figure 3E, on the 56th day of accelerated oxidation, the PV decreased in the order CK > TP > BHT > QG > PG. This showed that QG had good antioxidant activity and prevented the rancidity of soybean oil, and the effect of QG was obviously better than those of TP and BHT but slightly weaker than that of PG. In addition, the content of free fatty acids in soybean oil can be determined by measuring the acid value (AV) of the oil. As shown in Figure 3C, the AVs of the pure oil samples increased as the accelerated oxidation time was increased; however, only the AV measured during the latest accelerated oxidation period (i.e., after 56 days) showed a significant difference. As shown in Figure 3F, on the 56th day of accelerated oxidation, the AVs of the soybean oil samples treated with 200 ppm positive control and QG were significantly reduced compared with that of pure soybean oil after 56 days of accelerated oxidation, and the magnitude of AV decreased in the order CK > TP > BHT > QG > PG; these results were consistent with those for PV, which indicated that QG effectively retarded the oxidative rancidity of soybean oil, and the effect was even better than those of BHT and TP. Our results were consistent with data reported in other studies on the use of BHT and natural extracts to inhibit primary lipid oxidation.

### 2.5. GC-MS Studies of Fatty Acids Produced in Soybean Oil at the End of Accelerated Oxidation

**Figure 4:**
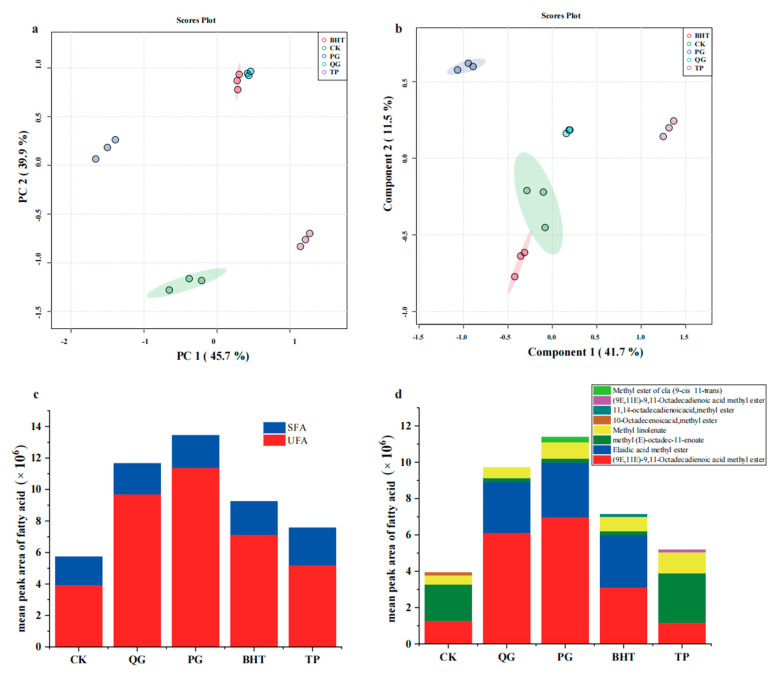
Fatty acid composition of oil treated with different antioxidants: PCA score plot (**a**); PLS-DA score plot (**b**); mean peak area of saturated fatty acids (SFAs) and unsaturated fatty acids (UFAs) (**c**); mean peak area of various unsaturated fatty acid (**d**).

This study involved analyses with different antioxidants (200 ppm QG, PG, BHT and TP) used to treat soybean oil. The fatty acid profiles of the analysed oils are shown in Table 3. Fourteen fatty acids (six saturated and eight unsaturated) were identified in total. The main unsaturated fatty acids (UFAs) were C18:1 (oleic acid) and C18:2 (linoleic acid). The saturated fatty acids were C10:0 (capric acid), C16:0 (palmitic acid), C18:0 (stearic acid), C20:0 (methyl arachidate) and C22:0 (behenic acid). Then, the data for total fatty acids (TFAs) were imported into the MetaboAnalyst 5.0 for further analyses, including PCA and PLS-DA. The PCA and PLS-DA results are shown in Figure 4a,b. PCA and PLS-AD showed good duplication within groups and significant differences among the five different groups of soybean oil samples. Figure 4c and Table 4 show the total amounts of saturated fatty acids (SFAs), unsaturated fatty acids (UFAs) and the ratio between unsaturated and saturated fatty acids (UFA/SFA) for the soybean oils analysed. The analytical results for fatty acid composition showed that the QG group had the highest proportion of unsaturated fatty acids, followed by the PG group, whereas the proportion of unsaturated fatty acids in TP was the lowest among the five groups. PG and QG had the highest proportions of UFAs, approximately 84.67% ± 0.33% and 82.94% ± 0.65% of total fatty acids (TFAs), respectively. Moreover, the UFA/SFA ratios were highest for the PG and QG groups and measured approximately 5.53 ± 0.14 and 4.87 ± 0.22, respectively. In contrast, there was no significant difference between the TP group and the CK group. The analytical results presented in Figure 4d and Table 4 showed that eight unsaturated fatty acids were detected in soybean oil, of which (9E,11E)-9,11-octadecadienoic acid exhibited the highest content, and the contents of the PG and QG groups were the highest and roughly five times more than that of the CK group. Otherwise, elaidic acid constituted a high proportion of unsaturated fatty acids but was only detected in the PG, QG and BHT groups. The above results showed that it is important to delay the oxidative rancidity of soybean oil and inhibit the oxidation of oleic acid and linoleic acid in unsaturated fatty acids, especially (9E,11E)-9,11-octadecadienoic acid and elaidic acid. Oxidation of QG during the process of delaying soybean oil oxidative rancidity provided a good antioxidant effect, which was significantly better than those for the positive controls BHT and TP but slightly weaker than that for PG. Moreover, the results showed the same trend seen for the peroxide values and acid values.

### 2.6. HS-SPME-GC-MS Studies of Volatiles in Soybean Oil at the End of Accelerated Oxidation

**Figure 5:**
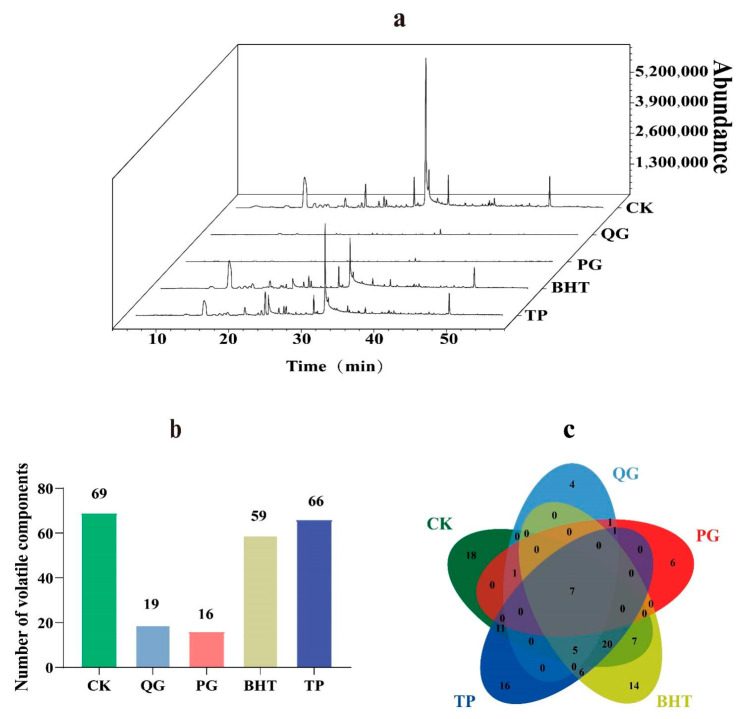
Total ion chromatograms (TIC) of soybean oil treated with different antioxidants via HS-SPME-GC-MS (**a**); the number of volatile components (**b**); Venn diagram of volatile components (**c**).

**Figure 6:**
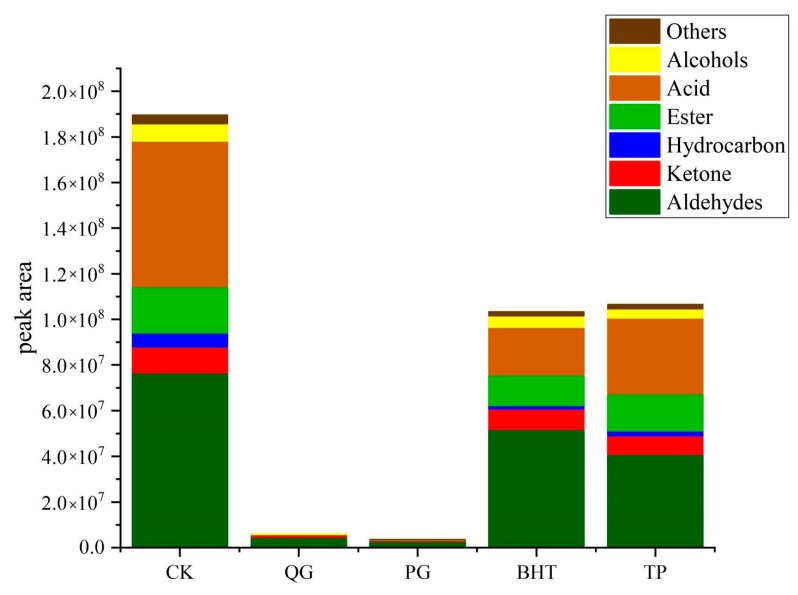
Peak area of various volatile fractions from soybean oil treated with different antioxidants according to HS-SPME-GC-MS.

To further understand the effect of QG on the oxidative stability of soybean oil subjected to accelerated oxidation, HS-SPME-GC-MS was used to analyse the volatile compounds (Table S2). Analyses of the volatile components in soybean oil identified 108 volatile components, including 16 alcohols, 23 aldehydes, 25 ketones, 4 acids, 15 esters, 18 hydrocarbons and 7 others (Figure 5a). Compared with the control group, the amounts and contents of volatile components were significantly reduced by antioxidant treatment. The most dramatic declines were seen for soybean oil treated with PG and QG. Specifically, the CK group contained 69 volatile constituents representing 63.9% of the total, while the QG and PG groups only exhibited 17.6% and 14.8% total volatile components (Figure 5b). Using a Venn diagram, we observed that these volatile components comprised seven components, including hexanal, octanal, trans-2-heptenal, (E)-2-octenal, butanoic acid, 2,2-dimethylethenyl ester, trans, trans-2,4-nonadienal and trans, trans-2,4-decadien-1-al, which were observed for all groups (Figure 5c). Additionally, the highest proportion of volatile components (46.36% of the total peak area) was found for the CK group, while the QG and PG groups only accounted for 1.45% and 0.87% of the total peak areas (Figure 6). In conclusion, the addition of QG retarded the production of volatile components in soybean oil and blocked the production of acids and alkanes. Moreover, the antioxidant capacity of QG was comparable to that of the known antioxidant (PG) but significantly better than those of BHT and TP. Most importantly, the trend for the degree of inhibition of volatile profiles was consistent with the trends for the peroxide value, acid value and fatty acid content.

## 3. Discussion

Frying has a long history as a classical and common process for preparing food, and fried foods have gained popularity due to the flavours and textures they exhibit. However, it is known that oils deteriorate during frying. Many chemical processes occur during heating, such as the oxidation of different fatty acids and triacylglycerols, the formation of polymers or cyclic compounds, the loss of volatile compounds and the formation of polycyclic aromatic hydrocarbons [42]. Moreover, the polyunsaturated fatty acid (PUFA) contents of vegetable oils decreases while those of saturated fatty acids (SFAs) usually increase. These changes in the chemical composition of heated oil pose serious risks to human health, such as hypertension [43], cardiovascular disease [44] and so on [45]. Antioxidants may protect the oil from the oxidation that occurs during storage and use, and the main role of antioxidants in oils is to protect unsaturated fatty acids against autoxidation. Plant-derived natural medications have been used for centuries and are becoming more popular because of their limited side effects. Several studies have reported that natural antioxidants, such as tocopherols [46], 2,4,4’-trihydroxychalcone [47] and curcuma [48], are effective in delaying oxidative rancidity in oils. In the present study, we have focused on the effect of QG on the peroxide value, acid value, volatiles profiles and fatty acid compositions of soybean oils and found significant changes in the volatile components and fatty acids during accelerated oxidation.

### 3.1. Effects of QG on Peroxide Value (PV) and Acid Value (AV)

PVs are used to detect peroxide formation in the early stages of lipid oxidation [49]. In this study, regular increases in PV as a function of the accelerated oxidation period were observed for all soybean oil samples after all time intervals, and these were attributed to the formation of primary oxidation products in the samples. Compared with that for pure soybean oil, the PV seen with QG use was significantly decreased, and the oxidation rate was also significantly decreased, which indicated that QG prolonged the initiation stage of soybean oil oxidation. Simultaneously, the antioxidant effect of QG was better than those of the positive controls BHT and TP, which may be because there are 6-OH groups in the aromatic ring of QG, while BHT has only one OH group, and TP is the general name for the polyphenols in tea. Studies have shown that quercetin, which is a nucleophile, delayed the formation of early lipid peroxidation products in oil [50]. Therefore, we speculate that QG is a polyhydroxy phenolic compound with a hydrogen-donating substituent on a benzene ring, since the antioxidant effects of phenolic compounds are related to their hydrogen-donating ability, that is, the number of ortho- and para-OH groups, the bond dissociation enthalpies (BDE) and the formation of hydrogen bonds [51]. QG provides more binding sites than quercetin, and QG can also function as a nucleophile; this was responsible for prolonging the initiation period of the oxidation reaction.

During oxidation, hydroxyl free radicals accumulate in large quantities, and long-chain polyunsaturated fatty acids react with free radicals to form alkanes, alcohols, ketones, aldehydes and finally, acids, which leads to rancidity in oils [52]. The free fatty acids in soybean oil were determined by measuring the acid value, which is the standard parameter for oil rancidity [53]. In this study, compared with the pure soybean oil, increases in the acid values of oil with QG and the positive control were inhibited during accelerated oxidation, which was consistent with the literature report [54]. QG reduced the content of free fatty acids formed during accelerated oxidation. It may be that QG is a flavonoid compound with various aromatic ring substituents and can serve as a hydrogen-donating antioxidant to delay the rancidity of soybean oil.

### 3.2. Effects of QG on Fatty Acid Content

Fatty acids are the main components of vegetable oils, including saturated, monounsaturated and polyunsaturated fatty acids, and polyunsaturated fatty acids are generally believed to be beneficial to human health [55]. The fatty acids in soybean oil mainly consist of oleic acid, linoleic acid, linolenic acid, palmitic acid and stearic acid, with linoleic acid having the highest content [56]. The results of our study showed that after the accelerated oxidation of soybean oil, the proportion of unsaturated fatty acids among all fatty acids was 69.06% ± 0.52%, while the unsaturated fatty acids in the QG group constituted 82.94% ± 0.65% of total fatty acids, indicating that QG significantly inhibited the oxidation of unsaturated fatty acids. More importantly, the polyunsaturated fatty acid (9E,11E)-9,11-octadecadienoic acid (C18:2) is related to linoleic acid, which exhibits the highest content in soybean oil, and the content in the QG group was approximately five times that of the control group. At the same time, the monounsaturated fatty acid elaidic acid (C18:1) belongs to oleic acid, and its content in soybean oil was second only to that of (9E,11E)-9,11-octadecadienoic acid. The content in the QG group was significantly higher than that of the control group. Both oleic acid and linoleic acid are 18-carbon unsaturated fatty acids, which have good nutritional and health care effects [57]. Oleic acid is a monounsaturated fatty acid with strong thermal stability; the carbon chain of linoleic acid is a polyunsaturated fatty acid containing two double bonds, and its thermal stability and antioxidant capacity are not as high as those of oleic acid [58]. Our results showed that linoleic acid accounted for the highest proportion among unsaturated fatty acids, the oxidation of linoleic acid was significantly inhibited by the addition of antioxidants, and the oxidation of oleic acid was also inhibited. It is speculated that the quercetin in marigolds delays oxidative rancidity of soybean oil by inhibiting the oxidation of the linoleic acid (9E,11E)-9,11-octadecadienoic acid methyl ester (C18:2) and the oleic acid elaidic acid methyl ester (C18:1).

### 3.3. Effects of QG on Volatiles Profiles

Under accelerated oxidation, oils first produce hydroperoxide, which is extremely unstable. After reaching a certain concentration in the oil system, it begins to decompose and form alkoxy radicals, and then forms hydrocarbons, alcohols, aldehydes, acids, etc. by different pathways; these exhibit odours, produce the so-called oily smell and eventually lead to rancidity of the oil [59,60]. We used headspace solid-phase microextraction–gas chromatography (HS-SPME-GC-MS) to qualitatively analyse the secondary oxidation products that result from the accelerated oxidation of soybean oil. The results showed that the oxidative rancidity of soybean oil resulted from secondary oxidation products, including aldehydes, ketones, esters, alcohols and alkanes, among which aldehydes accounted for the highest proportion. After peroxidation treatment, the contents and quantities of aldehydes were significantly reduced, which was consistent with literature reports [61]. In addition, the lipid cleavage reaction led to the formation of free fatty acids, thereby increasing the acidity of the oil; free fatty acids are one of the secondary oxidation products formed from acid compounds after oxidative rancidity of oil, and the addition of antioxidants can significantly inhibit the formation of acid compounds. Among these, the quercetin in marigolds completely blocked the formation of acid compounds [62]. Therefore, we believe that natural antioxidants can protect the oil from oxidation, avoid the formation of oily odour in the oil and, thus, prolong the shelf life.

## 4. Materials and Methods

### 4.1. Standards, Chemicals and Reagents

The acetonitrile, methanol and formic acid were of high-performance liquid chromatography (HPLC) grade and were purchased from Merck (Darmstadt, Germany). Deionized water was purified using a Milli-Q system (Millipore, Milford, MA, USA). The 2,2-Diphenyl-1-picrylhydrazyl (DPPH), butylated hydroxytoluene (BHT), tea polyphenols (TP) and propyl gallate (PG) were purchased from Aladdin (Aladdin Biochemical Technology Co., Ltd., Shanghai, China). The standards included quercetaget-in-7-O-glucoside (99%, CAS: 548-75-4, Batch No.: 19078-925) and quercetin (99%, CAS: 117-39-5, Batch No.: 190926-028), and they were obtained from Beijing Zhongxing Jiaren Biotechnology Co., Ltd. (Beijing, China). Quercetagetin (98%, CAS: 90-18-6, Batch No.: AF20022403) was obtained from the Beijing Beina Chuanglian Biotechnology Research Institute. The 0.22-µm pore organic phase filter membrane was purchased from Jinteng (Tianjin, China).

### 4.2. Preparation of Marigold Extract

Marigold was kindly provided by Hebei Chenguang Biotechnology Co., Ltd. (Handan, China). First, the marigold was ground in liquid nitrogen and lyophilized, and the sample powder was then extracted via material–liquid ratio of 1:10 with 95% methanol–water (v/v) and ultrasonicated for 2 h at 40 kHz, 100 W, and 25 °C using aKQ5200DE Ultrasonic cleaner (Kunshan, China). The total extract was centrifuged at 5000 rpm for 15 min at room temperature, and the supernatant was collected. Subsequently, the supernatant was passed through a 0.22 μm microporous filter membrane and transferred to HPLC vials for HPLC-Q-TOF-MS/MS and HPLC analysis.

### 4.3. DPPH and the Control Solution Preparation

The DPPH stock solution was prepared by weighing the appropriate amount of DPPH, and diluted solutions containing 0.625 mM, 1.25 mM, 2.5 mM and 5 mM DPPH free radical solutions with 95% methanol were prepared; the solutions were stored at 4 °C away from light. The quercetagetin-7-*O*-glucoside, quercetagetin and quercetin control were weighed precisely and dissolved in methanol to a mass concentration of 500 μg/mL, which were stored in a dark refrigerator at 4 °C.

### 4.4. Screening of the Potential Antioxidant Components in the Methanol Extract of Marigolds Using Offline DPPH-HPLC-Q-TOF/MS

Offline DPPH-HPLC-Q-TOF/MS was used to screen the potential antioxidant components in the methanol extract of marigold. Different concentrations of DPPH solution and marigold donors were thoroughly mixed in a 1:1 ratio, and the solution was stored in the dark and was incubated at 37 °C for 30 min. After that, the mixture was filtered through a 0.22 μm microporous filter membrane for HPLC analysis. The DPPH group was the mixture of DPPH solution and extract solution, and the DPPH-free group was the mixture of 95% methanol and the extract solution. The peak areas of the compounds with potential antioxidant activities in the HPLC chromatogram of the DPPH group were significantly lower than those of the DPPH-free group. The difference in the reduction in DPPH peak areas (PAs) between the DPPH-free sample and DPPH sample was used for determining the percent radical scavenging activity of the sample according to the following equation [56]:

$$\mathrm{Radical scavenging}\;(\%)=\frac{\mathrm{PAs}\;\mathrm{of}\;\mathrm{DPPH}-\mathrm{free}\;\mathrm{sample}\;-\;\mathrm{PAs}\;\mathrm{of}\;\mathrm{DPPH}\;\mathrm{sample})}{\left(\mathrm{PAs}\;\mathrm{of}\;\mathrm{DPPH}-\mathrm{free}\;\mathrm{sample}\right)\;}\;\times \;100$$

HPLC-Q-TOF/MS chromatography conditions: Chromatography was performed using an Agilent 1290 HPLC system (Agilent Technologies, Santa Clara, CA, USA) consisting of a rapid resolution binary pump, autosampler, thermostat column compartment, vacuum degasser and tuneable UV detector. Separation was carried out on an XAquaC18column (150 mm × 2.1 mm, 5 µm, 100 A; Acchrom Technologies Co., Ltd., Beijing, China). The elution system was 0.1% formic acid in water (A) and 0.1% formic acid in acetonitrile (B). The linear gradient elution program was as follows: 0−10 min, 5–25% B; 10−15 min, 25–35% B; 15−25 min, 35−70% B; 25−27 min, 70−90% B; 27−37 min, 90−95% B. The sample injection volume was 5 μL. The rate was set to 0.3 mL/min, and the column temperature was maintained at 30 °C.

### 4.5. Calibration of the Offline DPPH-HPLC-Q-TOF/MS Method

Propyl gallate (PG) was used as a positive control for calibration to evaluate the feasibility of the DPPH-HPLC-Q-TOF/MS method. Briefly, a certain concentration of PG solution was first prepared, i.e., processed according to the DPPH-HPLC-Q-TOF/MS antioxidant activity screening method. The DPPH group was a mixture of DPPH solution, extract solution and PG, whereas the DPPH-free group was a mixture of 95% methanol, the extract solution and PG, i.e., the positive control standard calibration, and the trends for peak area reductions in the positive control standard and the sample to be tested were used to determine the feasibility of the DPPH-HPLC method.

### 4.6. Identification of Potential Antioxidants in the Methanol Extract of Marigolds by HPLC-Q-TOF-MS/MS

To identify the screened antioxidants, HPLC-Q-TOF-MS/MS was used to determine their molecular weights. Mass spectrometry was performed using a 6530Q-TOF/MS accurate mass spectrometer (Agilent Technology, Santa Clara, CA, USA) in negative electrospray ionization mode, and TOF data were acquired between *m/z* 100 and 1000 in centroid mode. The Q-TOF/MS conditions were optimized as follows: drying gas, 10 L/min; fragmentation voltage, 150 V; sheath gas temperature, 350 °C; sheath gas flow, 11 L/min; gas temperature, 350 °C; capillary voltage, 4000 V; and skimmer voltage, 65 V. The TOF mass spectrometer was continuously calibrated using a reference solution to obtain high-accuracy mass measurements (masses at *m*/*z* 112.9855 and 966.0007 for negative). The target MS/MS experiments were performed using variable collision energy (10−40 eV), which was optimized for each individual compound.

### 4.7. Quantitative Analysis of the Identified Antioxidants in the Methanol Extract of Marigold Was Performed Using HPLC-PAD

External standard calibration was used for the quantitative analysis with slight modifications [63]. Mixed reference compounds were prepared with 95% methanol solutions. The master batch of reference compounds were diluted with methanol at concentrations of 500, 250, 125, 62.5, 31.25, 15.625, 7.8125 and 3.90625 µg/mL according to the multiplicative dilution method. After the reference solutions were prepared along a concentration gradient, they were injected into the HPLC column. The quantitative HPLC-PAD chromatography was performed on a Water Alliance E2695 Ultra HPLC Performance Liquid Chromatograph system (WATERS, Milford, MA, USA) equipped with an XAquaC18 column (150 mm × 2.1 mm, 5 µm, 100 A; Acchrom Technologies Co., Ltd., Beijing, China) and a photodiode array detector (PAD). The HPLC conditions included a flow rate of 0.3 mL/min at 30 °C, and the sample injection volume was set to10 μL. The elution system was acetonitrile (A) and 0.1% formic acid in water (B), and the solvent gradient was as follows: 0–15 min, 10–90% A; 16−20 min, 90–10%; 21−26 min, 10–10% A. The fraction was monitored at 359 nm. Standard curves of the reference compounds solution were made based on the linear relationship between the concentrations and peak areas (PAs). The amount of the identified antioxidants in the marigolds was calculated.

### 4.8. Peroxide Value and Acid Value

The effectiveness of QG in preventing the oxidation of soybean oil during accelerated oxidation was tested by using the Schaal oven heat resistance test method. Soybean oil with 100, 200, 400, 800 and 1600 ppm QG was added to soybean oil used for feeding, the control group (CK) was pure soybean oil and the positive control was soybean oil with 200 ppm BHT, PG and TP. Adjustments were made; samples from each group were collected at 0, 7, 14, 28 and 56 days, respectively; and PVs and AVs were determined with reference to the national standard methods GB5009.227-2016 and GB5009.229-2016, respectively.

### 4.9. Fatty Acids

#### 4.9.1. Methyl Esterification of Fatty Acids

Fatty acid methyl esters were prepared by the acid–base binding method. Assays were performed according to reference [64] with some modifications. Following the procedure previously described in detail: first, 10 mg of the oil sample was added to 1 mL of 1 mol/L KOH-methanol solution, and the mixture was shaken in a 40 °C water bath for 30 min. After shaking, it was left to stand for 15 min at room temperature. At the end of standing, the solution was placed in a 70 °C water bath to evaporate the solution to dryness. After evaporation of the solvent, 2 mL of 5% H_2_SO_4_/methanol (*w*/*v*) solution was added to the residue and shaken for 1 h at 70 °C in a water bath and then rapidly cooled to room temperature. After cooling ended, 2 mL of hexane was added, the mixture was shaken for 2 min, left standing for 10 min, and approximately 1.5 mL of the upper hexane extraction solution was placed into a 10 mL centrifugal tube. One g of anhydrous sodium sulfate was added, shaken for 1 min, left standing for 5 min, and the upper solution was placed into a sample bottle to be tested.

#### 4.9.2. Fatty Acid Composition and Content Analysis

Gas chromatography with a time-of-flight mass spectrometry system consisting of a Shimadzu GC-MS-QP2010 system (Shimadzu, Kyoto, Japan) with a fused silica HB-88 capillary column (100.0 m × 0.32 mm × 0.25 μm; Agilent, Santa Clara, CA, USA) was employed. Helium was used as the carrier gas (purity 99.999%); the inlet temperature was 25 °C, the initial column temperature was held at 120 °C for 1 min, raised at 10 °C/min to 175 °C, held for 10 min, raised at 5 °C/min to 210 °C, held for 5 min, raised at 5 °C/min to 230 °C, and held for 15 min. The column flow rate was 1.04 mL/min, the shunt ratio was 100, the ion source temperature was 200 °C, and the interface temperature was 220 °C. The scan range was 50~700 *m*/*z*, and the injection volume was 1 µL. The fatty acid compositions of the soybean oil were determined with the NIST 17 mass spectral library. The fatty acid content of the soybean oil was expressed as relative percentages for each fatty acid, which were calculated by external normalization of the chromatographic peak areas.

### 4.10. Characterization of Volatile Compounds

#### 4.10.1. Headspace Solid-Phase Microextraction (HS-SPME)

Soybean oil (3.00 ± 0.0001 g) was placed in a 20 mL vial with a manual headspace sampling system equipped with a 50/30 µm DVB/CAR/PDM fibre (Supelco, Bellefonte, PA, USA) [65]. Samples in headspace vials were pre-equilibrated at 100 °C for 40 min and extracted for 40 min at the same temperature. After extraction, the fibre was immediately inserted into the injection port of the GC-MS for thermal desorption at 240 °C for 5 min.

#### 4.10.2. Gas Chromatography–Mass Spectrometry (GC-MS) Analysis

Gas chromatography with a time-of-flight mass spectrometry system consisting of a Shimadzu GCMS-QP2010 system (Shimadzu, Kyoto, Japan) with a fused silica HB-88 capillary column (100.0 m × 0.32 mm × 0.25 μm; Agilent, Santa Clara, CA, USA) was employed. Injections were conducted with split-less mode at 240 °C. Chromatographic separations were performed as follows: 60 °C for 0–5 min, increased to 140 °C at a rate of 5 °C/min and held for 5 min, then increased to 210 °C at a rate of 5 °C/min and held for 10 min, then finally increased to 230 °C at a rate of 10 °C/min. Helium was used as the carrier gas with an average linear velocity of 1.37 mL/min. The mass spectrometer was operated with a transfer line temperature of 220 °C and an ion source temperature of 200 °C, and the electron impact ionization was tuned to 70 eV with masses ranging from 45 to 500 *m*/*z*.

### 4.11. Data Analysis

The data from the HS-GC-IMS and GC-MS were standardized using the SPSS software, and the results were plotted using Origin software. Principal component analysis (PCA) and Partial Least-Squares Discriminant Analysis (PLSDA) were used to evaluate similarities and differences with MetaboAnalyst 5.0.

## 5. Conclusions

Feed is susceptible to spoilage during processing, transportation and storage; this results in an unpleasant harsh taste, which is mainly caused by the oxidative rancidity of lipids in the feed. In this study, QG, a natural antioxidant with the highest content and strongest free radical scavenging ability, was screened and identified from forage marigold via offline DPPH-HPLC-Q-TOF-MS/MS. In addition, it was used as a natural antioxidant added to feed soybean oil and its effect in delaying the oxidative rancidity of the soybean oil was evaluated with different detection methods. These results showed that QG effectively reduced the peroxide value and acid value, decreased the formation of secondary oxidation products such as aldehydes, ketones and acids, etc., and inhibited the oxidation of unsaturated fatty acids such as oleic acid and linoleic acid. It also showed comparable or better effects than the positive control. Therefore, QG derived from the forage plant marigold can be used as a safe and effective natural antioxidant to stabilize feed soybean oil and extend the shelf life of feed by delaying the oxidative rancidity of soybean oil. This study provided valuable information for the development of natural antioxidants present in marigolds, and future studies should shed light on their antioxidation mechanisms.

## Figures and Tables

**Table 1:** Relative PAs of peak 1 (quercetagetin-7-*O*-glucoside), peak 2 (quercetagetin), peak 3 (quercetin) and peak 4 (patuletin) after reaction with different concentrations of DPPH.

		Relative PAs (%)			
No.	DPPH Concentration	Peak 1	Peak 2	Peak 3	Peak 4
1	Unreacted control sample	100	100	100	100
2	Reacted with DPPH (0.625 mM)	98.11 ± 1.02	81.75 ± 1.88	93.38 ± 2.45	93.42 ± 1.66
3	Reacted with DPPH (1.25 mM)	85.5 ± 1.68	14.42 ± 0.75	50.95 ± 1.68	61.17 ± 2.03
4	Reacted with DPPH (2.5 mM)	73.74 ± 0.93	3.65 ± 0.97	24.77 ± 1.21	13.34 ± 0.96
5	Reacted with DPPH (5 mM)	2.81 ± 0.06	0.47 ± 0.03	4.26 ± 0.47	12.37 ± 0.85

Note: Values are means ± SD (one-way ANOVA, *n* = 3, SPSS Statistics).

**Table 2:** Identified potential antioxidant compounds of the methanol extract of marigold.

Peak No.	tR (min)	Name	Formula	[M-H]- (*m*/*z*)	Error (ppm)	MS/MS Fragment Ions (*m*/*z*)
1	9.262	Quercetagetin- 7-*O*-glucoside	C_21_H_20_O_13_	479.0829	0.62	317.0304, 359.0403
2	12.144	Quercetagetin	C_15_H_10_O_8_	317.0303	1.57	139.0036, 166.9982, 149.0239, 121.0294, 111.0086, 271.0247
3	14.547	Quercetin	C_15_H_10_O_7_	301.0371	7.28	151.0039, 178.9998, 121.0296
4	15.748	Patuletin	C_16_H_12_O_8_	331.0449	−1.51	316.0216, 165.9896, 209.0081

Note: error (ppm) = (Measured *m*/*z* values−theoretical *m*/*z* values)/theoretical *m*/*z* values × 10^6^.

**Table 3:** Individual fatty acid composition in soybean oil (mean ± SD, range).

No.	Chemical Name		Mean Peak Area of Fatty Acids (×10^6^)				
			CK	QG	PG	BHT	TP
1	(9E,11E)-9,11-Octadecadienoic acid methyl ester	C18:2	1.27 ± 0.12 d	6.11 ± 0.44 b	6.97 ± 0.28 a	3.1 ± 0.045 c	1.16 ± 0.14 d
2	Elaidic acid methyl ester	C18:1	ND	2.82 ± 0.08 a	3.03 ± 0.08 a	2.9 ± 0.49 a	ND
3	methyl (E)-octadec-11-enoate	C18:1	2.01 ± 0.39 b	0.21 ± 0.01 c	0.21 ± 0.46 c	0.21 ± 0.35 c	2.74 ± 0.29 a
4	Methyl linolenate	C18:1	0.5 ± 0.04 c	0.57 ± 0.11 c	0.9 ± 0.33 ab	0.8 ± 0.14 bc	1.16 ± 0.14 a
5	10-Octadecenoicacid,methyl ester	C18:1	0.15 ± 0.02	ND	ND	ND	ND
6	11,14-Octadecadienoicacid, methyl ester	C18:2	ND	ND	ND	0.12 ± 0.015	ND
7	(9E,11E)-9,11-Octadecadienoic acid methyl ester	C18:2	ND	ND	ND	ND	0.12 ± 0.01
8	Methyl ester of cla (9-cis, 11-trans)	C18:2	0.06 ± 0.03 b	ND	0.27 ± 0.07 a	ND	ND
9	Behenic acid methyl ester	C22:0	0.05 ± 0.01 a	0.063 ± 0.006 a	0.05 ± 0.01 a	0.0367 ± 0.06 b	0.057 ± 0.01 a
10	Methyl palmitate	C16:0	1.17 ± 0.19 b	1.3 ± 0.04 ab	1.41 ± 0.1 ab	1.42 ± 0.23 ab	1.48 ± 0.13 a
11	Methyl stearate	C18:0	0.51 ± 0.07 b	0.58 ± 0.03 ab	0.6 ± 0.03 ab	0.61 ± 0.11 ab	0.65 ± 0.06 a
12	Caprylic acid methyl ester	C8:0	ND	ND	ND	ND	0.06 ± 0.005
13	Methyl arachidate	C20:0	ND	0.047 ± 0.005 a	ND	0.04 ± 0.017 a	0.047 ± 0.01 a
14	Dimethyl azelate	C10:0	0.07 ± 0.01 a	ND	ND	ND	0.09 ± 0.005 b

Note: “ND” indicates not detectable. Values are means ± SD (one-way ANOVA, *n* = 3, SPSS Statistics). Significant differences between diet groups at the indicated week are signified by letters, where different letters indicate difference (*p* < 0.05) between groups, while the same letter indicates no difference.

**Table 4:** Saturated fatty acids (SFAs), unsaturated fatty acids (UFAs), unsaturated and saturated fatty acids (UFA/SFA) ratio, unsaturated and total fatty acids (UFA/TFA) of analysed soybean oils. Results are reported as peak area percent ± uncertainty expressed as the half-width of the 95% confidence interval.

No.	Fatty Acid	CK	QG	PG	BHT	TP
1	UFA (peak area (×10^6^)	4.02 ± 0.48 e	9.72 ± 0.60 b	11.42 ± 0.5 a	7.27 ± 0.48 c	5.17 ± 0.56 d
2	SFA (peak area (×10^6^)	1.8 ± 0.24 b	2.0 ± 0.04 b	2.07 ± 0.1 ab	2.11 ± 0.35 ab	2.3 ± 0.2 a
3	UFA/SFA	2.2 ± 0.5 d	4.87 ± 0.22 b	5.53 ± 0.14 a	3.48 ± 0.33 c	2.15 ± 0.53 d
4	UFA/TFA (%)	69.06% ± 0.52% d	82.94% ± 0.65% b	84.67% ± 0.33% a	77.58% ± 0.17% c	68.30 ± 0.535% d

Note: Values are means ± SD (one-way ANOVA, *n* = 3, SPSS Statistics). Significant differences between diet groups at the indicated week are signified by letters, where different letters indicate difference (*p* < 0.05) between groups, while the same letter indicates no difference.

## Data Availability

Not applicable.

## Supplementary Materials

The following supporting information can be downloaded at: https://www.mdpi.com/article/10.3390/molecules27092865/s1, Figure S1: Mass spectra of marigold flower extract peak 1, peak 3 and peak 4; Figure S2: HPLC chromatograms of a standard solution containing the three reference compounds (a) and the methanolic extract of marigold (b). Table S1: Calibration curve parameters of three antioxidants identified in the marigold; Table S2: Relative content of volatile compounds identified in soybean oil treated with different antioxidants under accelerated oxidation.

## Sample Availability

Samples of the compounds are not available from the authors.
