# Supplementary material for: Effect of Natural Antioxidants from Marigolds (Tagetes erecta L.) on the Oxidative Stability of Soybean Oil
Source: Molecules. 2022 Apr 30;27(9):2865. doi: 10.3390/molecules27092865 (PMC9105600; doi:10.3390/molecules27092865)
Supplement: Supplementary file 1 [file molecules-27-02865-s001.zip › molecules-1701791-supplementary.pdf]

Effect of Natural Antioxidants from Marigolds（*Tagetes erecta* L.）on the Oxidative Stability of Soybean Oil

Supplementary Data

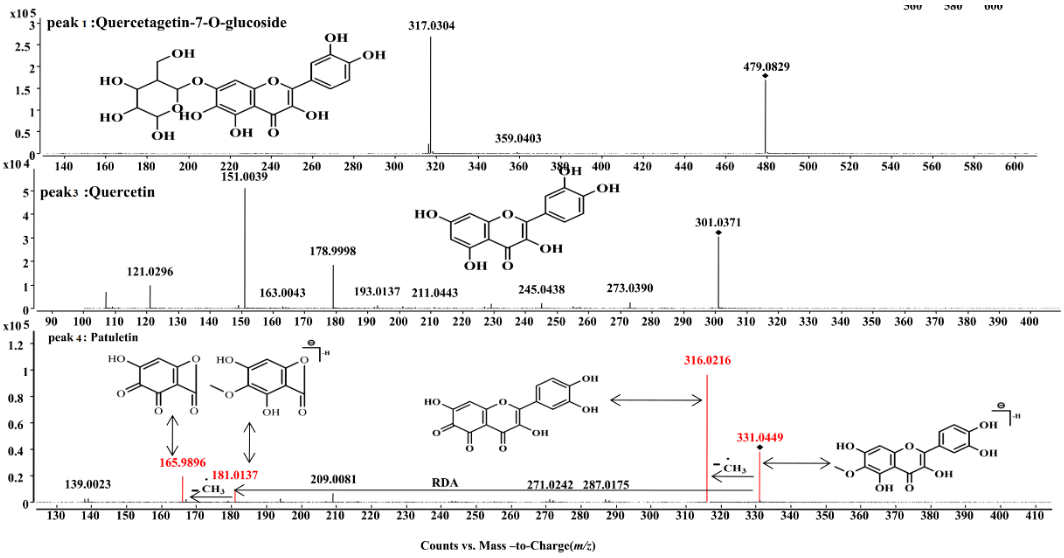

Figure S1. Mass spectrum of Marigold flower extract peak 1, peak 3 and peak 4.

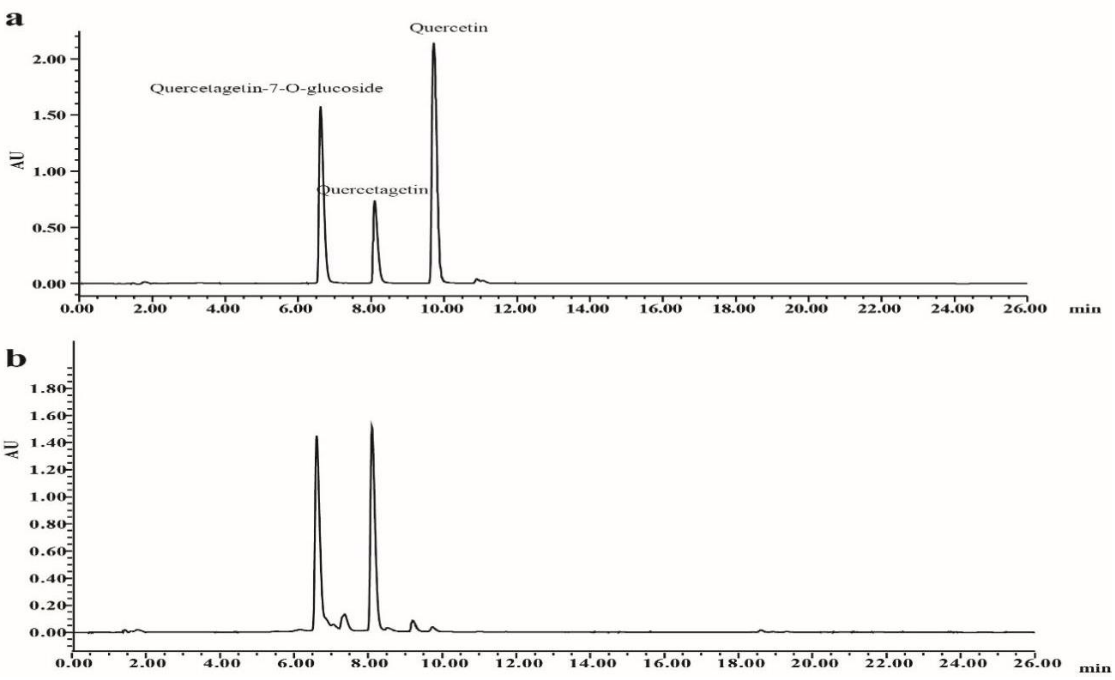

Figure S2. HPLC chromatograms of a standard solution containing the three reference compounds (**a**) and the methanolic extract of marigold (**b**).

Table S1. Calibration curve parameters of three antioxidants identified in the marigold

Compound	Calibration curve	Correlation coefficient	Linear range (μg/mL)
Quercetagetin-7-O-glucoside	Y = 38951X + 68479，	0.999848	3.90625-250
Quercetagetin	Y = 70372X + 16577	0.998933	3.90625-500
Quercetin	Y = 138041X - 44724	0.999985	3.90625-125

Table S2. Relative content of volatile compounds identified in different antioxidants treated soybean under accelerated oxidation

			Relative content（%）				
**NO.**	**tR(min)**	**Name of compound**	**CK**	**QG**	**PG**	**BHT**	**TP**
1	8.284	Heptane	2.26	-	-	-	-
2	8.942	Octane	0.13	-	-	-	-
3	12.363	Pentanal	1.61	-	-	2.59	1.77
4	14.927	Hexanal	18.55	19.65	4.27	30.24	15.38
5	16.344	Furan, 2-pentyl	1.91	-	-	1.06	0.73
6	16.993	1-Pentanol	1.1	-	-	-	1.51
7	17.007	1-Butanol, 3-methyl	-	-	-	1.63	-
8	17.351	5-Hexen-2-one, 5-methyl-3-methylene	-	9.62	-	3.7	-
9	45.868	1-Octene, 3,4-dimethyl	0.43	-	-	0.44	0.53
10	17.711	Heptanal	1.12	-	-	-	2.47
11	18.140	2-Heptanone	1.23	-	-	2.89	1.73
12	19.519	Acetic acid	1.25	-	-	-	0.42
13	19.644	Butanoic acid, 3-methyl	-	-	-	1.40	-
14	20.079	Formic acid, heptyl ester	-	-	-	-	0.22
15	20.515	Octanal	2.47	2.38	-	3.09	3.51
16	20.823	1-Methoxy-3-hydroxymethylheptane	-	-	-	0.57	-
17	20.843	2-Heptanone, 5-methyl	0.41	-	-	-	0.50
18	21.168	3-Octanol	-	-	-	-	0.29
19	22.048	4-Nonanone	-	-	-	1.21	1.51
20	22.112	3-Octanone, 2-methyl	0.12	-	-		
21	22.333	1-Octen-3-ol	0.45	1.26	-	0.88	1.09
22	22.782	2-Heptenal, (E)	1.09	5.49	4.48	1.45	1.09
23	23.665	2-Nonanone	0.18	-	-	-	-
24	24.091	2,3-Dimethyl-3-heptene, (Z)	0.18	-	-	-	-
25	24.397	3-Ethylcyclopentanone	0.08	-	-	0.36	-
26	24.790	5-Octen-2-ol, 5-methyl	0.26	-	-	-	-

27	24.790	1,2-Epoxynonane	-	-	-	0.31	-
28	24.809	5-Decanone	-	-	-	-	0.270
29	25.162	3,5-Octadien-2-ol	1.09	-	-	1.47	
30	25.187	Hexanoic acid, pentyl ester	-	-	-	-	1.670
31	25.872	2-Octenal, (E)	2.02	3.66	4.09	3.16	1.94
32	26.196	Decanal	1.12	-	-	1.7	2.03
33	26.567	2-Decanone	-	-	-	0.23	0.32
34	26.924	Cyclopropanemethanol, .alpha.-butyl	0.17	-	-	-	0.090
35	27.543	6-Undecanone	0.4	-	-	0.4	0.61
36	27.718	2,4-Heptadienal, (E,E)	4.75	3.18	6.55	-	-
37	27.768	cis-4-Decenal	-	-	-	0.18	-
38	28.214	trans-3-Nonen-2-one	0.67	-	-	0.65	0.54
39	28.352	2,4-Heptadienal, (E,E)	-	10.02	4.79	-	0.07
40	28.776	6-Undecanol	-	-	-	-	0.100
41	28.927	2-Nonenal, (E)	0.37	-	-	0.35	0.3
42	29.155	3,5-Octadien-2-one, (E,E)	-	2.56	3.48		
43	30.324	6-Dodecanone	0.13	-	-	0.41	0.35
44	30.513	Butyric acid, 4-pentadecyl ester	4	2.07	2.85	4.69	4.96
45	30.527	2-Oxo-n-valeric acid	0.5	-	-	-	-
46	30.830	1-Nonen-4-ol	0.18	-	-	0.08	
47	31.565	Hexanoic acid	32	-	-	18.87	30.83
48	31.988	2-Decenal, (E)	3.31	2.4	-	2.63	7
49	32.546	Hexane, 3-methyl-4-methylene	0.19	-	-	0.19	-
50	32.875	Hexanoic acid, octyl ester	-	-	-	-	0.550
51	32.898	6-Tridecanone	0.27	-	-	0.32	
52	33.231	n-Caproic acid vinyl ester	0.69	-	-	0.64	0.65
53	33.418	Hexane, 2,2,3,3-tetramethyl	-	-	-	-	0.330
54	33.422	2,2-Dimethyl-3-heptanone	-	-	-	0.36	-
55	33.450	Acetyl valeryl	0.31	-	-	-	-
56	33.833	(S)-(+)-5-Methyl-1-heptanol	0.22	-	-	-	0.390
57	34.174	Hexanoic acid, 2-propenyl ester	0.14	-	-	-	-
58	34.363	2,4-Nonadienal, (E,E)	0.09	2.26	7.69	0.12	0.09
59	34.371	2,4-Nonadienal, (E,E)	-	-	2.01	-	-
60	34.626	2-Undecenal		-	2.08	-	-
61	34.663	2-Undecenal	3.1	2.34	20.93	1.33	1.25
62	34.910	Dodecane, 1-iodo	-	-	-	-	0.500
63	35.352	6-Tetradecanone	0.1	-	-	0.78	0.940
64	35.686	Tetrahydrofurfuryl acrylate	0.08	-	-	-	-
65	36.265	2,4-Decadienal, (E,E)	0.08	6.27	4.16	0.38	0.31
66	37.082	Cyclooctane, 1,4-dimethyl-, trans	-	-	20.93	-	-
67	37.097	2,4-Decadienal	0.21	17.38	2.33	1.14	1.180
68	37.602	Docosane	-	-	-	0.12	-
69	37.691	6-Pentadecanone	0.08	-	-	-	0.17

70	38.020	1,3-Cyclohexanedione, 2-methyl	0.2	-	-	0.32	-
71	38.106	Pentanoic acid, 2-methyl-, anhydride	0.04	-	-	0.21	-
72	39.400	2-Pentadecanone	0.16	-	-	0.15	0.270
73	39.518	Tetratetracontane	-	-	-	0.10	
74	39.785	C2yclopentanecarboxylic acid, 2-tetrahydr	-	-	-	0.16	-
75	39.802	2,5-Dimethylcyclohexanol	0.12	-	-	-	0.15
76	40.381	2(3H)-Furanone, 5-ethyldihydro	0.59	-	-	-	-
77	40.665	4-Oxohex-2-enal	0.32	0.58	-	0.37	0.330
78	40.776	4-Oxononanal	0.25	-	-	0.17	0.2
79	41.018	cis-4,5-Epoxy-(E)-2-decenal	-	-	4.16	-	
80	41.037	Cyclohexanone, 2-ethyl	0.97	5.45	-	0.78	1.07
81	41.308	2-Acetonylcycloheptanone	0.08	-	-	-	
82	41.310	Heneicosane	-	-	-	-	0.360
83	41.666	Hexadecanoic acid, ethyl ester	-	-	-	-	0.150
84	41.981	Hexanal ethyl trans-2-hexenyl acetal	-	-	-	-	0.220
85	41.986	Hexanal ethyl trans-2-hexenyl acetal	-	-	-	0.18	-
86	42.006	Acetaldehyde di-trans-2-hexenyl acetal	0.08	-	-	-	-
87	42.768	2(3H)-Furanone, dihydro-5-propyl	0.06	-	-	-	0.090
88	43.047	Tetracosane	-	-	-	0.13	0.270
89	43.421	Cyclohexadecanone	-	-	-	0.11	
90	43.433	2-Heptadecanone	0.1	-	-		
91	44.029	4-Heptenal	0.13	-	-		0.320
92	44.034	cis-4,5-Epoxy-(E)-2-decenal	-	-	-	0.22	
93	44.428	Hexanethioic acid, S-heptyl ester	0.09	-	-	-	0.160
94	44.659	Triazaadamantane	0.1	-	-	-	-
95	44.737	Triacontane	-	-	-	0.04	-
96	44.830	2-Decen-1-ol, (E)	-	-	-	0.21	-
97	44.844	trans-2-Dodecen-1-ol	0.15	-	-	-	-
98	45.542	2(3H)-Furanone, 5-butyldihydro	0.14	-	-	0.13	0.180
99	46.524	Octacosane	-	-	-	0.24	0.180
100	48.267	2(3H)-Furanone, dihydro-5-pentyl	0.34	-	-	0.35	0.330
101	48.438	Hentriacontane	-	-	-	0.08	0.09
102	48.650	E-2-Hexenyl E-2-octenoate	4.49	1.71	-	5.51	5.55
103	50.583	Pentacosane	-	-	-	-	0.130
104	50.687	2(3H)-Furanone, dihydro-4-methyl-5-pentyl	0.07	-	-	-	-
105	52.538	1-Dodecyne	0.07	-	-	-	-
106	52.540	2-Methyltetracosane	-	-	-	0.09	0.11
107	53.955	3-Decyn-2-ol	0.19	-	-	-	0.110
108	54.575	Hexatriacontane	-	-	-	-	0.040
